# Nitrate decreases methane production also by increasing methane oxidation through stimulating NC10 population in ruminal culture

**DOI:** 10.1186/s13568-017-0377-2

**Published:** 2017-04-04

**Authors:** Lihui Liu, Xiurong Xu, Yangchun Cao, Chuanjiang Cai, Hongxiao Cui, Junhu Yao

**Affiliations:** College of Animal Science and Technology, Northwest A&F University, Yangling, 712100 Shaanxi People’s Republic of China

**Keywords:** Ruminal methane emission, Nitrate, Ammonium salt, Denitrifying anaerobic methane oxidizing, NC10

## Abstract

Studies proved that addition of nitrate in rumen could lead to reduction of methane emission. The mechanism of this function was involved in the competition effect of nitrate on hydrogen consumption and the inhibitory effect of generated nitrite on methanogen proliferation. The present study investigated an alternative mechanism that denitrifying anaerobic methane oxidizing (DAMO) bacteria, DAMO archaea and anammox bacteria may co-exist in rumen, therefore, more methane can be oxidized when addition of nitrate. Ruminal batch culture model was used to test the effects of addition of 5 mM NaNO_3_, 4 mM NH_4_Cl, or both into the culture substrate on methane production, fermentation patterns, and population of methanogens, NC10 and anaerobic methanotrophic-2d (ANME-2d). Our results showed that NC10 in the ruminal culture was detected by polymerase chain reaction (PCR) when using NC10 special primer sets, and addition of nitrate reduced methane production and the relative proportions of methanogen, whereas increased the relative proportion of NC10. A combined addition of ammonia salt and nitrate did not show further inhibitory effect on methane production but accelerated nitrate removal. We did not detect DAMO archaea in ruminal culture by real-time PCR when using ANME-2d special primer sets. The present study may encourage researchers to pay more attention to methane oxidation performed by anaerobic methanotroph when studying the strategies of inhibiting ruminal methane emission.

**Electronic supplementary material:**

The online version of this article (doi:10.1186/s13568-017-0377-2) contains supplementary material, which is available to authorized users.

## Introduction

There are increasing evidences showing the significant inhibitory effect of nitrate on methane production in vitro and methane emission from the rumen in vivo (Zhou et al. 2012; Patra and Yu 2014; Elzaiat et al. 2014; Olijhoek et al. 2016). An acceptable mechanism of this effect is that the reduction of nitrate or nitrite consumes hydrogen, which reduces hydrogen available for methane formation in the rumen (Ao and Emeritus 2008; Nolan et al. 2010). Nitrite is the intermediate product in nitrate reduction process. A detrimental problem of dietary supplementation of nitrate to ruminants is that nitrite could be accumulated in the rumen and then absorbed into the circulating system through the rumen wall, and results in a change of hemoglobin to methemoglobin (Sar et al. 2004), which is incapable of carrying oxygen and causes mild to severe methaemoglobinaemia. The toxic character of nitrite to ruminants leads to the controversy of nitrate supplementation, even though there are increasing proofs that nitrite would not accumulate to the toxic level in the rumen of sheep acclimated to nitrate supplementation.

Guo et al. (2009) reported that the percentage of nitrogen to the total gas production was markedly increased when addition of nitrate into the ruminal fermentation system. In another study, the product of ammonia after an administration of nitrate was much lower than expected (Tillman et al. 1965), which was unlikely to be the major cause of the disappearance of nitrate (Ao and Emeritus 2008), indicating that there should be other metabolic pathways of nitrate in rumen.

Research in the field of environmental protection has shown that nitrate can be reduced to nitrite by ANME-2d, and nitrite can be subsequently converted to nitrogen by ‘*M. oxyfera*’, which belongs to NC10 phylum. To date, NC10 bacteria are the only known bacteria that can anaerobically oxidize methane; all other anaerobic methanotrophs are archaea (He et al. 2016). ANME-2d and ‘*M. oxyfera*’ are two anaerobic methanotrophs and exist ubiquitously in the anaerobic environment abundant in methane, such as freshwater sludge, landfill and wastewater (Wu et al. 2012). The denitrification process of nitrate and nitrite coupled to methane oxidation is named as denitrifying anaerobic methane oxidation (DAMO), and believed as a potential process to remove nitrate and nitrite in wastewater even the DAMO rate is very low in vitro culture. It had been reported that DAMO can be extremely accelerated in an anaerobic ammonium oxidation reactor because nitrite produced from nitrate reduction can be jointly reduced to nitrogen when ammonium is oxidized to nitrogen by anammox (Chen et al. 2015). Therefore, nitrite produced from nitrate has two pathways to be converted to nitrogen in the mixture reactor of both DAMO and anaerobic ammonium oxidation when provided with nitrate, ammonium salt and methane simultaneously, and nitrite reduced from nitrate in the mixed reactor can be cleaned more rapidly than in the reactor with DAMO system alone. Actually, anammox also ubiquitously exists. The combined pathways of DAMO and anaerobic ammonium oxidation in the sludge of freshwater were proposed by Haroon et al. (2013).

The ruminal microorganisms can adapt to addition of nitrate (Alaboudi and Jones 1986; Zhou et al. 2012), anaerobic oxidation of methane has been detected in the rumen (Valdés et al. 1996; Kajikawa et al. 2003) and an inclusion of nitrate in ruminal fermentation in vitro decreases methane production while increases nitrogen production (Guo et al. 2009). These facts together led to a generation of our research hypothesis that DAMO bacteria, DAMO archaea and anammox may exist in rumen. The present study tested this hypothesis by investigating the effect of addition of both nitrate and NH_4_Cl on methane production and nitrite accumulation compared with an addition of either nitrate or NH_4_Cl in in vitro ruminal fermentation, meanwhile anammox, DAMO bacteria and archaea were also detected by PCR.

## Materials and methods

### Experimental design

Batch culture was used to examine the effect of nitrate and NH_4_Cl on ruminal methane production and fermentation characters. There were four treatments: addition of 5 mM sodium nitrate (NaNO_3_), addition of 4 mM ammonium chloride (NH_4_Cl), addition of 5 mM NaNO_3_ and 4 mM NH_4_Cl, and the control. There were three replicates for each treatment.

The ruminal fluid was used as the inoculum for batch culture. The fluid was collected from three fistulated Xinong Saanen dairy goat at approximately 8:30 after morning feeding. The fluid from the three goats was pooled at equal proportion, and immediately filtered through four layers of sterile cheesecloth. The culture medium consisted of artificial saliva and the rumen fluid at a 2:1 ratio. The artificial saliva was prepared according to the formula provided by Menke et al. (1979). The batch culture was carried out in 200-mL culture bottles with 0.4 g ground alfalfa as the fermentation substrate and 60 mL of the culture medium. Before incubation, the bottles were vacuumed and then inflated with CO_2_. The incubation was conducted at 39 °C for 24 h in a simulative incubator (ANKOM DAISY II, Ankom Technology, Macedon, New York, USA).

### Sampling and chemical analysis

After 24 h incubation, the fermentation was terminated by placing bottles on ice. The volume of the total gas produced in the culture bottles was determined using glass syringe, and the concentrations of CH_4_, CO_2_ and H_2_ in the total gas were determined in gas chromatography (Machmuller et al. 2003). The pH value of the culture was immediately measured using a pH meter. The content of each bottle was filtered through filter bags (Ankom Technology, Macedon, New York, USA) and the residue was used to determine degradability of the feed substrate. The filtrate was aliquoted into different centrifuge tubes, and parts of each aliquot stored at −80 °C for measurement of volatile fatty acids, microflora and the concentrations of NO_3_^−^ and NO_2_^−^. The concentrations of volatile fatty acids (VFA) were measured by gas chromatography according to Zhao et al. (2010). The aliquot for the analysis of ammonia was added with sulphuric acid (1%, w/v), and the ammonia concentration was determined using a colorimetric method (Chaney and Marbach 1962). The concentrations of NO_3_^−^/NO_2_^−^ were determined by colorimetric methods (Lambert and Zitomer 1960; Wang et al. 2015). The degradability of dry matter (DM), neutral detergent fiber (NDF) and acid detergent fiber (ADF) of the substrate were analyzed using the methods described by van Soest et al. (1991).

### DNA extraction

Metagenomic DNA in 3 mL of the culture fluid was extracted using modified cetyltrimethyl ammonium bromide (CTAB) method (Kumar et al. 2014). The quality of the extracted DNA was assessed using agarose gel (1%) electrophoresis and the concentration of DNA was determined in absorbance at 260 and 280 nm using a NanoDrop ND2000 spectrophotometer (NanoDrop Technologies Inc., DE, USA). The DNA samples were stored at −20 °C until analysis.

### Determination of the bacterial number by quantitative real-time PCR

The population size of total bacteria in the samples was determined by SYBR green-based absolute quantitative real-time PCR (qPCR). To prepare the qPCR DNA standard, a general PCR was performed using the total bacteria specific primers set (Patra and Yu 2014). The purified PCR products were then cloned into the PMD19-T vector (TaKaRa Biotechnology, Dalian, China) and transformed into the competent cells of DH5α. One of the positive clones was cultured, and then the recombined DNA of the plasmid in the cultured positive clone was extracted by TIANprep Mini plasmid Kit (Tiangen, Beijing, China) and used as the qPCR DNA standard. The concentration of this DNA standard was diluted to 30 ng/μL and then was subject to a serial of tenfold dilutions to make the calibration curve (Patra and Yu 2014). The copy number of the diluted DNA standard was calculated based on the mass concentration and the average molecular weight of the recombined DNA following the equation described by Yu et al. (2005). The number of total bacteria in each sample with 30 ng metagenomic DNA could be calculated from the calibration curve according to its Ct value and was used to deduce the number of total bacteria in one mL ruminal culture by multiplying the original volume of the extracted DNA and its dilution multiple.

The relative proportions of NC10, methanogens, anammox bacterium and ANME-2d to the total number of bacteria were determined by quantitatively competitive PCR. The relative proportion values were calculated using the 2^−ΔΔCt^ method as previously described (Zhu et al. 2014).

The reaction mixture (20 μL) of real-time PCR consisted of 10 μL of SYBR Premix Ex Taq (Takara Biotechnology (Dalian) Co., Ltd, Dalian, Liaoning, China), 0.4 μM of each primer and 30 ng (10-fold dilutions of 30 ng, for the DNA standard in absolute qPCR) of the extracted bacterial genomic DNA. The qPCR assay for each sample was performed in triplicate for both the diluted DNA standards and bacterial DNA with the same master PCR plate and reaction mixture. Real-time PCR in the present study was performed on Bio-rad-IQ5 PCR System (BioRad, Laboratories Inc., Hercules, CA, USA). The primers used for detecting the bacteria are listed in Table 1.

**Table 1:** The specific primers for PCR and qPCR

Target organisms	Primer	Primer sequence (5′–3′)	References
Total bacteria	Forward^b^	ACTCCTACGGGAGGCAGCAGT	Schwiertz et al. (2010)
	Reverse^b^	ATTACCGCGGCTGCTGGC	
Methanogens (mcrA)	Forward^b^	AACGCTAGCTACAGGCTT	Denman et al. (2007)
	Reverse^b^	CCAATGTGGGGGACCTTC	
NC10	qP1mF^b^	GGGCTTGACATCCCACGAACCTR	He et al. (2016)
	qP1R^b^	GCGTCAAGCTGGAGGAAGGCG	
Anammox	Pla46F^a^	GGATTAGGCATGCAAGTC	Amano et al. (2007)
	AMX1480R^a^	TACGACTTAGTCCTCCTCAC	
	amoA-1F^b^	GGGGTTTCTACTGGTGGT	Li et al. (2009)
	amoA-2R^b^	CCCCTCKGSAAAGCCTTCTTC	
ANME-2d	DP142F^a^	TAATACYGGATAGATCAAAG	Ding et al. (2015)
	DP779R^a^	GCACCGCACCTGACACCT	
	DP397F^b^	TGGCTGTCCAGCTRTYC	
	DP569R^c^	GRACGCCTGACGATTRAG	

^a^Primers used for general PCR

^b^Primers used for qPCR

^c^Primers used for general PCR and qPCR

### Statistical analysis

The data were analyzed in one-way ANOVA procedure (SPSS 19.0) to test the differences among four treatments in ruminal fermentation characters and the population of the total bacteria, methanogens and NC10. The least square means and standard error of means are presented. Significance was declared at *P* < 0.05 or *P* < 0.01, and trends were discussed at *P* < 0.1.

## Results

### Effects of treatments on production of the total gas, methane and hydrogen

Compared with the control (Table 2), addition of NaNO_3_ or NaNO_3_ + NH_4_Cl significantly reduced the volume of the total gas and methane over the 24 h incubation (*P* < 0.01), whereas inclusion of NH_4_Cl alone reduced the total gas only by 5.3% (*P* < 0.05) and did not change methane production (*P* > 0.05). The volume of hydrogen was lowered when addition of NaNO_3_ or NaNO_3_ + NH_4_Cl (*P* < 0.05).

**Table 2:** Effects of treatments on the volume of the total gas, methane and hydrogen (mL)

	Treatments				SEM	*P* value
	Control	NaNO_3_	NH_4_Cl	NaNO_3_ + NH_4_Cl		
Total gas	56.75^A^	36.25^D^	53.75^B^	48.00^C^	2.045	<0.001
CH_4_	12.99^Aa^	7.37^Bb^	13.00^Aa^	7.59^Bb^	0.722	<0.001
H_2_	0.02^b^	0.03^a^	0.02^b^	0.03^a^	0.002	0.049

Values with different lowercase or capital superscripts in the same row differ significantly at *P* < 0.05 and *P* < 0.01, respectively

### Effects of treatments on ruminal fermentation characters

As showed in Table 3, addition of NaNO_3_, NH_4_Cl or NaNO_3_ + NH_4_Cl obviously increased concentration of ammonia nitrogen (*P* < 0.01), but didn’t affect pH value, total VFA and acetate concentrations in the fermentative culture (*P* > 0.05) when compared with control; addition of NaNO_3_ or NaNO_3_ + NH_4_Cl decreased butyrate concentration (*P* < 0.01).

**Table 3:** Effects of treatments on fermentative characters in ruminal culture

	Treatments				SEM	*P* value
	Control	NaNO_3_	NH_4_Cl	NaNO_3_ + NH_4_Cl		
pH	6.77	6.83	6.83	6.82	0.013	0.302
Ammonia (mg/dL)	20.83^Cb^	23.52^Ba^	22.29^BCa^	26.13^A^	0.623	<0.001
Acetate (mM)	21.67	26.54	22.34	24.40	0.934	0.264
Propionate (mM)	4.92	4.55	5.12	4.28	0.180	0.369
Isobutyrate (mM)	0.43	0.38	0.35	0.30	0.032	0.579
Butyrate (mM)	1.96^Aa^	1.21^Bb^	2.07^Aa^	1.36^Bb^	0.122	0.002
Isovalerate (mM)	0.52	0.65	0.54	0.47	0.043	0.580
Valerate (mM)	0.38	0.42	0.38	0.33	0.021	0.549
Total volatile fatty acids (mM)	29.87	33.75	30.80	31.13	1.035	0.651

Values with different lowercase and capital superscripts in the same row differ significantly at *P* < 0.05 and *P* < 0.01, respectively

Table 4 showed that nitrate concentration in the ruminal culture was increased by addition of NaNO_3_ (*P* < 0.01), but not by addition of NaNO_3_ + NH_4_Cl, neither by addition of NH_4_Cl (*P* > 0.05). Inconsistent with the nitrate concentration, the nitrite concentration was not different among the four treatments.

**Table 4:** Effects of treatments on the degradability of DMD, and nitrate and nitrite concentrations in ruminal culture

	Treatments				SEM	*P* value
	Control	NaNO_3_	NH_4_Cl	NaNO_3_ + NH_4_Cl		
DMD (%)	54.60	53.54	54.22	52.93	0.368	0.422
NDFD (%)	46.57	48.39	46.73	49.12	0.423	0.053
ADFD (%)	32.40	33.44	32.27	34.03	0.396	0.372
Nitrate (mM)	0.74^Ba^	1.94^A^	0.82^Ba^	0.92^Ba^	0.150	<0.001
Nitrite (mM)	0.16	0.18	0.16	0.13	0.007	0.183

Values with different lowercase and capital superscripts in the same row differ significantly at *P* < 0.05 and *P* < 0.01, respectively

The degradability of DM and ADF remained similar among the four treatments (*P* > 0.05), but NDF degradability tended to be increased by addition of NaNO_3_ and by NaNO_3_ + NH_4_Cl (*P* < 0.1).

Addition of NaNO_3_ or NaNO_3_ + NH_4_Cl significantly increased the proportion of acetate to total VFA (*P* < 0.01), whereas decreased the proportion of propionate to total VFA (*P* < 0.01) (Table 5). The proportion of butyrate to total VFA was decreased by NH_4_Cl and NaNO_3_ + NH_4_Cl (*P* < 0.01, *P* < 0.05) but not by NaNO_3_ alone (*P* > 0.05).

**Table 5:** Effects of treatments on the proportions of individual VFA in vitro rumen fluid culture

	Treatments				SEM	*P* value
	Control	NaNO_3_	NH_4_Cl	NaNO_3_ + NH_4_Cl		
Acetate (%)	72.56^Bb^	78.66^Aa^	72.43^Bb^	78.42^Aa^	0.983	0.001
Propionate (%)	16.53^Aa^	13.48^Bb^	16.66^Aa^	13.68^Bb^	0.484	<0.001
Isobutyrate (%)	1.28	1.12	1.13	0.92	0.085	0.589
Butyrate (%)	6.60^Aa^	6.75^Aa^	3.59^Bb^	4.51^ABb^	0.465	0.006
Isovalerate (%)	1.75	1.92	1.77	1.43	0.122	0.617
Valerate (%)	1.28	1.24	1.26	1.03	0.056	0.426
Acetate/propionate	4.40^Bb^	5.85^Aa^	4.35^Bb^	5.74^Aa^	0.228	<0.001

Values with different lowercase and capital superscripts in the same row differ significantly at *P* < 0.05 and *P* < 0.01, respectively

### Effects of treatments on microbial populations

As shown in Table 6, addition of NaNO_3_, NH_4_Cl or both did not alter the number of total bacteria in the culture (*P* > 0.05). Supplementation of NaNO_3_ alone or together with NH_4_Cl decreased the relative proportion of methanogen in total bacteria compared with control (*P* < 0.05), and increased the relative proportion of NC10 in total bacteria compared with control and NH_4_Cl groups (*P* < 0.05). Addition of NH_4_Cl alone did not affect the relative proportion of NC10 in total bacteria (*P* > 0.05).

**Table 6:** Effects of treatments on the number of total bacteria and the relative proportion of methanogen and NC10 in total bacteria

	Treatments				SEM	*P* value
	Control	NaNO_3_	NH_4_Cl	NaNO_3_ + NH_4_Cl		
Methanogen	0.99^a^	0.78^b^	0.93^a^	0.70^b^	0.040	0.007
NC10	0.98^b^	1.25^a^	1.07^b^	1.26^a^	0.040	0.005
Total bacteria (log copies/mL fluid)	9.79	9.49	9.58	9.77	0.079	0.373

The proportion of methanogen or NC10 in total bacteria was detected by relative quantification real-time PCR (Zhu et al. 2014), therefore there was no unit for this index. Values with different lowercase superscripts in the same row differ significantly (*P* < 0.05)

We investigated ANME-2d and anammox bacteria using ANME-2d and anammox bacterium special primers PCR, but did not detect any of them either in the fresh ruminal fluid or in the ruminal fluid culture.

## Discussion

We found in the current experiment that an addition of 5 mM nitrate, alone or with ammonium salt, decreased methane production in the ruminal culture and changed the fermentative pattern with an increase of acetate percentage, but decrease of butyrate and propionate percentages. The total VFA production and the degradability of DMD were unaffected by treatments. The effect of nitrate on methane production in ruminal culture in the present study was in line with other reports (Zhou et al. 2012; Patra and Yu 2014; Elzaiat et al. 2014; Olijhoek et al. 2016).

The inhibitory effect of nitrate on methane production in rumen is believed via a mechanism that the reduction process of nitrate and nitrite to NH_4_ competes with CO_2_ for hydrogen (Zijderveld et al. 2010; Patra and Yu 2014). The increased NH_4_ concentration in response to addition of nitrate in our study supported this mechanism. Another mechanism of the inhibitory effect of nitrate on methane production is the toxic function of nitrite (the first intermediate of nitrate reduction) on methanogens (Božic et al. 2009; Zhou et al. 2011) and could be confirmed by the result in the current study that the relative population of methanogens in total bacteria was significantly decreased by nitrate addition. However, we noticed that the nitrite concentration after 24 h incubation was unchanged in response to nitrate addition. It was reported that nitrate/nitrite in the rumen fluid was detectable at early stage with an administration of nitrate into the rumen, but undetectable after about 8 h (Wang et al. 1961). Therefore, it was suspected that nitrite concentration in the ruminal culture might be increased at the beginning of the experiment as a result of addition of nitrate, but the differences in nitrite concentration among the treatments diminished after 24 h incubation because nitrite could be further reduced. If so, the inhibition of nitrite on methanogen proliferation might occur at early stage, but this inhibition had a sustained reductive effect on the number of methanogens in the ruminal culture.

It was noticed in this study that nitrate was accumulated in response to nitrate addition in the ruminal culture, and the nitrate concentration in nitrate group was 1.2 mM higher than that in control group after 24 h incubation. This result is inconsistent with previous in vivo study (Wang et al. 1961). Ao and Emeritus (2008) proposed the possible reasons for rapid clearance of nitrate/nitrite in rumen, including absorption of nitrate into the host’s blood. The reason why there is a difference in the change of nitrate concentration between the present study and other in vivo experiments is probably because that there was no absorptive pathway for nitrate in an in vitro culture system. However, when nitrate was added with ammonium salt together, no increase in nitrate concentration was observed and indicated that more nitrate was removed compared with addition of nitrate alone. Alaboudi and Jones (1986) reported that some rumen bacteria could acclimate to nitrate addition. In addition, there were evidences proving that methane oxidation was coupled to nitrate or nitrite reduction by DAMO archaea and bacteria in sludge of lake or river (Haroon et al. 2013; Chen et al. 2015), and the removal of nitrite could be accelerated after DAMO was coupled to the anammox bacterial system (Chen et al. 2015). The bacteria that can reduce nitrite to nitrogen in this mixed system include NC10 and anammox bacteria (Hu et al. 2015). Rumen is a methane enrichment habitat and is connected with the external environment through the digestive tract, and DAMO bacteria and anammox bacteria may also exist in rumen. The detectable NC10 in ruminal culture in this study partly supported this hypothesis. The fact that addition of nitrate increased the NC10 population indicated that NC10 was one of the bacteria that can acclimate to nitrate addition.

A combination of nitrate and ammonium salt in the present study resulted in more nitrate removal but no more methane reduction. A possible explanation was that anammox bacteria in the rumen culture could consume more nitrite as a result of addition of ammonia and hence accelerated nitrate reduction, but the nitrite reduction pathway performed by anammox didn’t increase the oxidized methane, therefore, there is no change in methane production compared with addition of nitrate alone. However, anammox bacteria in ruminal culture were not detected by common or real-time PCR in the current study. But Candidatus Kuenenia, an identified anammox bacterium, was found in rumen fluid when the ruminal bacterial flora was investigated by high through-put sequencing of the 16s rRNA gene (unpublished). It’s worth noting that Candidatus Kuenenia is the anammox species present in the enrichment culture of fresh water sediment (Haroon et al. 2013). The reason for no detectable anammox by anammox special primers PCR in this study was possibly because of its low abundance in rumen fluid. Actually, only ten sequences among the sequenced 27, 736 PCR products belong to Candidatus Kuenenia (Additional file 1). The fact that ANME-2d was not detected by PCR when using ANME-2d special primers in our study suggested that ANME-2d, the DAMO archaea, may not exist in rumen fluid.

In conclusion, NC10 could be detected in rumen fluid by NC10 special primers PCR. The present study indicated addition of nitrate in the culture reduced methane production not only by inhibiting methanogens but also by stimulating NC10 population. A combination of nitrate and ammonia salt had no further inhibitory effect on methane production, but could accelerate nitrate removal in vitro. ANME-2d wasn’t detectable in rumen fluid by ANME-2d special primers PCR.

## Additional file

**Additional file 1.** Unpublished sequencing data.

## Abbreviations

DAMO: denitrifying anaerobic methane oxidizing
PCR: polymerase chain reaction
ANME: archaea anaerobic methanotrophic-2d
DM: dry matter
NDF: neutral detergent fiber
ADF: acid detergent fiber
VFA: volatile fatty acids
CTAB: cetyltrimethyl ammonium bromide
