# Supplementary material for: Nitrate decreases methane production also by increasing methane oxidation through stimulating NC10 population in ruminal culture
Source: AMB Express. 2017 Apr 4;7:76. doi: 10.1186/s13568-017-0377-2 (PMC5380647; doi:10.1186/s13568-017-0377-2)

The following screenshot is Part of our sequencing data. The red-marked genus is Candidatus Kuenenia, which is one of the anammox speiceses in freshwater sediment. The total seqeunced PCR product in sample Y00 is 27, 736.


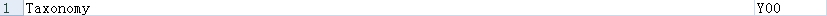


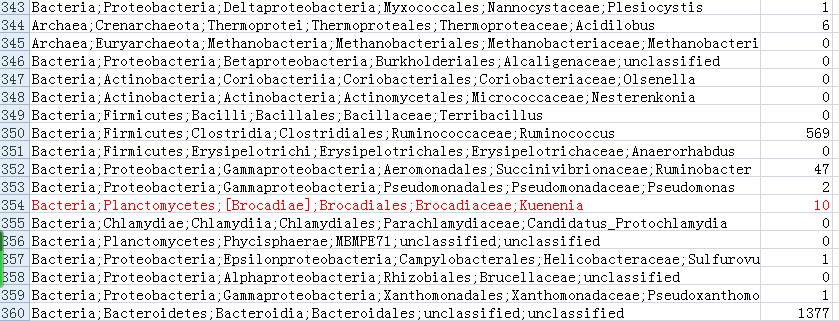


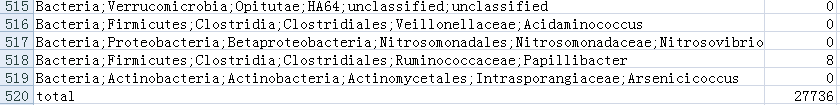

Supplement: Supplementary file 1 — Additional file 1. Unpublished sequencing data. [file 13568_2017_377_MOESM1_ESM.docx]
